# Limits to compensatory adaptation and the persistence of antibiotic resistance in pathogenic bacteria

**DOI:** 10.1093/emph/eou032

**Published:** 2014-12-21

**Authors:** R. Craig MacLean, Tom Vogwill

**Affiliations:** Department of Zoology, University of Oxford, South Parks Road, Oxford, OX1 3PS, UK

**Keywords:** antibiotic resistance, fitness cost, compensatory adaptation, experimental evolution, clinical microbiology

## Abstract

Antibiotic resistance carries a fitness cost that could potentially limit the spread of resistance in bacterial pathogens. In spite of this cost, a large number of experimental evolution studies have found that resistance is stably maintained in the absence of antibiotics as a result of compensatory evolution. Clinical studies, on the other hand, have found that resistance in pathogen populations usually declines after antibiotic use is stopped, suggesting that compensatory adaptation is not effective *in vivo*. In this article, we argue that this disagreement arises because there are limits to compensatory adaptation in nature that are not captured by the design of current laboratory selection experiments. First, clinical treatment fails to eradicate antibiotic-sensitive strains, and competition between sensitive and resistant strains leads to the rapid loss of resistance following treatment. Second, laboratory studies overestimate the efficacy of compensatory adaptation in nature by failing to capture costs associated with compensatory mutations. Taken together, these ideas can potentially reconcile evolutionary theory with the clinical dynamics of antibiotic resistance and guide the development of strategies for containing resistance in clinical pathogens.

## COMPENSATORY ADAPTATION AND THE MAINTENANCE OF ANTIBIOTIC RESISTANCE

Evolving antibiotic resistance by horizontal gene transfer or by chromosomal mutation is associated with a fitness cost, as demonstrated by the fact that resistant bacteria have reduced growth rates and competitive ability relative to sensitive strains in the absence of antibiotics [1–5]. Because resistance carries a cost, stopping antibiotic use once resistance begins to spread in a bacterial population should provide a simple and effective strategy for preventing the emergence of resistance [5–9]. In a classic experiment, Schrag and Perrot [10] tested this idea by allowing populations founded by streptomycin-resistant clones of *Escherichia coli* to evolve in the absence of antibiotics. Contrary to their expectations, they found that resistance was stably maintained in the absence of antibiotics because resistant clones adapted to the cost of resistance by evolving second-site compensatory mutations that recovered the cost of resistance [11]. A large number of studies have now used the same basic experimental design developed by Schrag *et al.* to study how bacteria adapt to the cost of resistance (reviewed in Ref. 2). They have utilized a wide range of study organisms, including major human pathogens such as *Mycobacterium tuberculosis, Staphylococcus aureus, E. coli, Streptococcus pneumoniae* and *Pseudomonas aeruginosa*. These studies have also used a variety of antibiotics from at least eight different antibiotic classes, as well as several mobile genetic elements (MGEs) that carry antibiotic resistance genes. These studies have reported that resistance, regardless of species or antibiotic, is almost always maintained in the absence of antibiotics as a result of compensatory adaptation [2, 4, 12].

The best evidence for compensatory adaptation in natural populations comes from studies that have found evidence for known compensatory adaptations in clinical isolates. For example, an early study by Nagaev *et al.* [13] found that clinical fusidic acid-resistant strains of *S.aureus* carry mutations in domains of EF-G that have shown to be involved in compensatory adaptation in the laboratory. More recently, targeted and whole-genome sequencing of clinical isolates has shown that adaptation to the cost of rifampicin resistance has occurred in isolates of *M.tuberculosis* [14–16]. However, despite the chronic nature of tuberculosis (TB) infections and high rates of person-to-person transmission, these studies have found compensation in a minority of cases—approximately 30% of global drug-resistant isolates. Although these elegant studies clearly show that compensatory adaptation is possible in natural pathogen populations, they do not provide any general insights into the prevalence of compensatory adaptation.

If compensatory adaptation is commonplace in clinical bacterial pathogens, then resistance should be maintained after antibiotic use is discontinued. A simple way to test for the maintenance of antibiotic resistance is to measure the prevalence of resistant isolates in samples taken from patients before and after exposure to antibiotics relative to suitable controls. A recent meta-analysis of this type of study performed on community infections concluded that the frequency of resistant isolates increases following antibiotic treatment, and then slowly returns to towards the baseline level [17]. For example, the frequency of macrolide resistance in streptococci rapidly increased following treatment with azythromicin or clarithromycin, but resistance declined back to near baseline levels by 12 months post-treatment [18]. More dramatically, β-lactam resistance in the *Haemophilus* populations of children who were treated with amoxicillin declined back to baseline levels within 12 weeks [19].

At a broader scale, it has been shown that resistance follows repeatable seasonal fluctuations that lag behind antibiotic prescription rates [20, 21]. For example, prescriptions for antibiotics in the US peak in the winter and then decline towards a low in the summer. Resistance also follows this pattern, but with a lag. These seasonal cycles are repeated across years, so that the frequency of resistance towards commonly used antibiotics remains relatively constant [20]. Clearly, this pattern is not what you would expect if compensation was rampant. It is important to note that reducing antibiotic consumption does not necessarily result in reduced antibiotic resistance. For example, large-scale reductions in the use of sulphonamide and trimethoprim to treat *E.coli* infections at a regional or national scale had little or no impact on the frequency of antibiotic resistance [22, 23]. In both of these cases, resistance did not carry a cost in the laboratory [23, 24], raising the possibility that these interventions may have failed in part because of the fact that compensatory adaptation had removed the cost of resistance prior to reductions in antibiotic use. However, genotypes carrying sulphonamide and trimethoprim resistance determinants tended to also be resistant to the antibiotics that were used to treat *E.coli* infections once the use of sulphonamide and trimethoprim were discontinued. Therefore, the role of compensatory adaptation in these interventions remains speculative. More generally, a recent meta-analysis of more than 100 studies examining spatial and temporal associations between antibiotic resistance and consumption concluded that there was a strong association between consumption and resistance [25]. If compensatory adaptation is common, then resistance should be only weakly coupled to consumption level, and the strong association that exits between consumption and resistance therefore provides support for the idea that compensatory adaptation is rare in clinical settings.

A second way to test for compensatory adaptation in clinical settings is to compare the fitness of co-occurring isolates that are antibiotic sensitive and resistant. Given that resistance carries a cost, sensitive and resistant isolates should tend to have equal fitness if compensatory adaptation is widespread. To test this, we searched the published literature for studies which had compared the fitness of co-occurring susceptible and resistant clinical isolates from the same species. Fitness could be measured by either direct competition experiments, where isolates are co-cultured, or by indirect methods, such as comparisons of maximum growth rate in mono-cultures. Studies that have used this approach have usually found that resistant isolates have a lower average fitness than sensitive isolates (Box 1 [26–38]). For example, this approach has shown that methicillin-resistant isolates of *S.aureus* (MRSA) from the first MRSA epidemic in Northern Europe had lower fitness than methicillin-sensitive strains, and this could explain why MRSA rapidly declined when methicillin use was reduced [39]. It is important to emphasize that these studies are associated with several caveats. For example, these studies do not control for genetic background and fitness is measured in the laboratory, rather than in a natural environments. Nonetheless, these studies clearly support the idea that compensatory adaptation is unable to maintain resistance in clinical settings.

**Box 1. Fitness of antibiotic resistant clinical isolates**

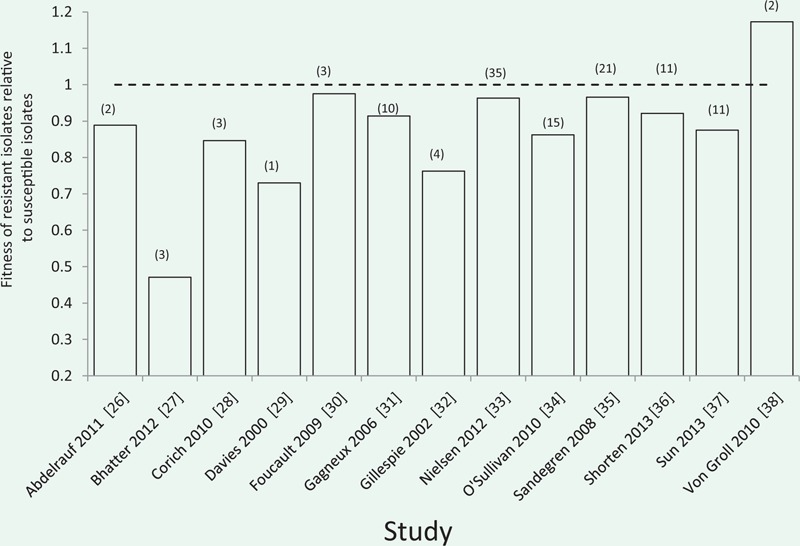

This graph summarizes the results of studies that have compared the fitness of co-occuring resistant and sensitive isolates in the absence of antibiotics, such that fitness = 1 represents equal competitive ability. Numbers in brackets represent the number of resistant isolates examined in each study. Fitness was estimated by either direct competition between sensitive and resistance isolates, or by proxy methods such as comparing rates of exponential growth. Resistant isolates have reduced competitive ability in 12/13 studies, and this difference is highly significant under a binomial test (*P* < 0.001). These studies were identified as part of a systematic meta-analysis of laboratory estimates of the cost of resistance [47]. However, these studies were not included in the meta-analysis presented in [47], clinical isolates can differ in more than just the resistant determinant.

In summary, laboratory studies find that bacteria have the ability to adapt to the cost of antibiotic resistance by compensatory mutation, suggesting that resistance should be maintained in the absence of antibiotics. Clinical studies, on the other hand, find that resistance usually declines when antibiotic use is stopped or discontinued, and resistant strains usually have lower fitness than conspecific-sensitive strains. These results suggest that selection for compensatory adaptation is not able to stabilize resistance in natural populations of pathogenic bacteria.

## LIMITS TO COMPENSATORY ADAPTATION

### Sensitive strains survive antibiotic treatment

One feature of laboratory studies of compensatory adaptation is that selection lines are always initiated with a single, antibiotic-resistant clone. In most of these experiments, resistance is caused by a single chromosomal mutation, and it is highly improbable that resistance will revert using this experimental design because of the rarity of reversion mutations. Single base pair substitutions in bacteria arise at a rate of approximately 10^−^^10^ to 10^−^^11^ per genome per generation [40], implying that even large bacterial populations will have to wait a very long time before reversion mutations occur. For example, in a population of 10^6^ cells a reversion mutation is only expected to occur once every 10 000 generations. Moreover, the majority of reversion mutations will be lost due to genetic drift when they are rare, even if resistance carries a large cost [41]. Given these simple considerations, it is remarkable that some studies have found evidence for the loss of resistance as a result of the spread of *de novo* reversion mutations [13, 42–44]. The reversion of resistance is also unlikely in populations initiated with clones carrying plasmid-borne resistance genes. Plasmids can be spontaneously lost at cell division, potentially generating a source of antibiotic-sensitive revertants. However, many plasmids possess high-fidelity partitioning mechanisms that ensure even segregation of plasmids at cell division and post-segregational killing mechanisms which efficiently eliminate cells lacking plasmids [45]. Consequently, although estimates of the rate of segregational loss of plasmids are lacking, it is generally accepted that the effective rate of segregational loss of most plasmids is very low. The horizontal transfer of plasmids by conjugation may also help to maintain resistance in the absence of selection. In populations of bacteria carrying conjugative plasmids, cells that lose plasmids by segregational loss risk being re-infected with plasmids by conjugation, and conjugation could theoretically allow costly plasmids to persist indefinitely [46].

By initiating populations with a single-resistant clone, experimental evolution studies of compensation effectively assume that antibiotic treatment eliminates all antibiotic-sensitive cells a population. In natural populations this assumption is unlikely to hold. First, there is heterogeneity in antibiotic exposure within patients and this heterogeneity is likely to generate refuges containing relatively low doses of antibiotic [48]. Second, there is heterogeneity in antibiotic sensitivity between genetically identical cells of ‘sensitive’ strains. The classic example of this heterogeneity is persister cells: a small fraction of cells in sensitive strains which are completely refractory to even very high doses of antibiotics because they are metabolically dormant [49, 50]. Recent work has shown that phenotypic heterogeneity in clonal bacterial populations extends far beyond persistence [51–53]. For example, variation in the expression of genes implicated in resistance ensures that some cells in sensitive strains have elevated resistance [54–56]. We argue that the consequence of variation in antibiotic exposure coupled to variation in antibiotic resistance ensures that a small fraction of cells that lack mutations or mobile elements conferring high levels of resistance are likely to survive exposure to treatment.

**Figure 1.**
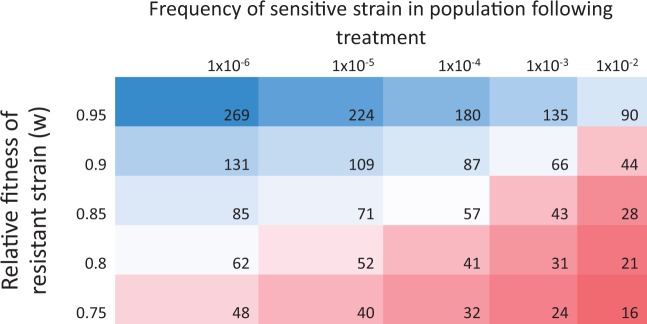
Half-life of resistance following antibiotic treatment This figure shows the expected half-life (measured in number of generations) of an antibiotic resistant strain (R) in competition with a higher fitness antibiotic sensitive strain (S) following antibiotic treatment. The key prediction of the model is that resistance should be rapidly eliminated in the absence of antibiotics, provided that sensitive strains make up a small fraction of the population that survives treatment. Half-life was calculated from log (*S_t_*/*R_t_*) = log (*S*_0_/*R*_0_) + *t* log (*w*) where *t* is time and *w* is the relative fitness of the resistant strain. We assume that half-life is the time taken for the resistant strain to decline to a frequency of 0.5.

Given that resistance carries a cost and that a small fraction of sensitive cells are likely to survive treatment with antibiotics, then resistance will only be able to persist if the resistant population is able to adapt to overcome the cost of resistance before being displaced by sensitive competitors. To better understand this process, we used a simple population genetic model of competition between a resistant and sensitive strain of bacteria. We assume that resistance carries a fixed cost, and that the sensitive strain makes up a non-zero fraction of the bacterial population after treatment (Fig. 1). The insight of this model is that resistance is likely to be lost very quickly, even if only a small fraction of the population that survives antibiotic treatment is antibiotic sensitive. For example, the average cost of a chromosomal resistance mutation is approximately 20% [47], and the model predicts that an average resistance mutation will be rapidly lost, even if only a small fraction of the population that survives treatment is sensitive. The quantitative predictions of this model need to be taken with a grain of salt; however, because it ignores the pharmacodynamics of antibiotic exposure [48] and it also does not consider the possibility of compensatory adaptation. One important exception to this argument is that the survival of sensitive strains is unlikely to constrain compensation in patients suffering from chronic infections. Patients suffering from chronic infections, such as those associated with cystic fibrosis and TB, are given extended treatments with antibiotics that can last for months. Under these extreme conditions, sensitive strains are likely to eradicated, and this process may help to explain why some of the best evidence for compensatory adaptation is from *M.tuberculosis* [14–16, 57].

### Imperfect compensation drives reversion

Compensatory mutations increase the fitness of resistant strains, but it has often been observed that compensatory mutations do not completely recover the cost associated with resistance [11, 43, 58]. We speculate that compensatory mutations provide an imperfect solution to the cost of resistance because compensatory mutations are associated with costs of their own. In support of this idea, compensatory mutations are often found in essential genes [2, 59], and it is well established that compensatory mutations are costly in the absence of resistance mutations [11, 43, 58]. The fact that compensation is imperfect is unlikely to matter in standard laboratory selection experiments, as strains carrying compensatory mutations will still have a fitness advantage relative to their resistant competitors. There is also some evidence to suggest that compensatory mutations are less effective at recovering the cost of resistance under natural conditions than under laboratory conditions. Bjorkman *et al.* [43] found that compensation was less likely for fusidic acid resistance in *Salmonella typhymurium* when passaged through a mouse as opposed to laboratory media. Crucially, all compensatory mutations, whether selected *in vitro* or *in vivo*, were more effective at restoring fitness *in vitro* than *in vivo.* It therefore appears that even when compensatory mutations fully restore laboratory fitness, this compensation is environment specific and therefore may not be as effective in clinical environments. Unfortunately, this study was not able to determine why compensatory mutations were unable to effectively restore fitness *in vivo*, and it is therefore unclear to what extent the results of this study are generally applicable to antibiotic resistance. Future experimental work should focus on measuring the fitness effects of compensatory mutations *in vivo*, and determining how environmental and genetic variation influence the benefits associated with compensatory adaptation.

## CONCLUSION

In an influential article published in 2010, Andersson and Hughes [2] argued that the conditions favouring the loss of established resistance genes in populations of pathogenic bacteria are likely to be very restrictive. Although there are some elegant examples of compensatory adaptation in clinical pathogens that support this view [13, 14, 16, 60–62], recent clinical studies show that resistance is usually lost in pathogen populations once antibiotic resistance is reduced or stopped. In contrast, laboratory experiments have almost always found that resistance is maintained in the absence of antibiotics. In this article, we argue that this discrepancy arises because there are limits to compensatory adaptation in nature that are not captured by the design of current laboratory selection experiments. In clinical settings, there is good empirical evidence that antibiotic treatment will often fail to eradicate ‘sensitive’ bacteria, and there are good theoretical reasons for thinking that surviving antibiotic-sensitive strains should rapidly replace resistant strains that are inferior competitors. In contrast, laboratory experiments favour compensatory adaptation, because compensatory mutations carry large benefits in the laboratory, and the design of laboratory experiments protects resistant clones carrying compensatory mutations from competition with high fitness antibiotic-sensitive clones.

Antibiotic resistance has been demonstrated to carry a fitness cost, both *in vitro* and *in vivo* [2]. However our understanding of the importance of compensatory adaptation in mitigating this cost *in vivo* remains fragmentary because of a lack of synergy in the approaches used to study the maintenance of resistance. Experimental evolution studies provide detailed insights into the dynamics and mechanisms of compensatory adaptation, but under conditions that lack several key features of the natural biological context in which resistance evolves. For example, experimental evolution studies have focused on using model systems involving a single chromosomal resistance mutation, whereas antibiotic-resistant clinical isolates usually carry multiple resistance mutations or MGEs that provide resistance against a broad spectrum of antimicrobials. Experimental evolution studies should place a greater emphasis on studying clinically relevant resistance mechanisms, and it would be especially interesting to see future work on resistance genes that pose the most important threats to human health, such as MGEs carrying carbapenem resistance genes [63–65]. On average, MGEs carry a smaller fitness costs than chromosomal resistance mutations, and this may help to explain why mobile elements have played such an important role in the evolution of resistance in human pathogens [65–68]. Experimental evolution studies have shown that compensatory adaptation can eliminate the cost of MGEs [69–71], but it is unclear if it is easier to evolve compensatory adaptations for MGEs than chromosomal mutations. When resistance evolves by MGE acquisition, compensatory adaptations can occur on both the chromosome and on the MGE [69], and this may accelerate compensatory adaptation. An important assumption of this argument is that compensation benefits both bacteria and their MGEs, which is likely to be the case for mobile elements that have predominantly vertical transmission, such as non-mobilizable plasmids and integrative and conjugative elements (ICEs). It is more difficult to predict the impact of compensatory adaptation on the fitness of MGEs with a high rate of horizontal transmission, such as conjugative plasmids and lysogenic bacteriophages. For example, trade-offs in the cost imposed by MGEs on alternative hosts may restrict the evolution of compensatory adaptations that improve fitness on a single host.

Another important difference between experimental evolution studies and clinical settings comes from bacteria that are used as model organisms. Experimental evolution studies tend to use well-characterized strains of important pathogens, such as *E.coli*, *S.aureus* and *M.tuberculosis*. These strains are usually highly domesticated, and can differ substantially from the dominant clones of these bacteria that cause infections in clinical settings. It is unclear to what extent the use of model strains has biased the outcome of laboratory studies, but a recent study that measured the cost of rifampicin resistance across strains of *Pseudomonas* shows that genetic background plays a dominant role in determining the cost of resistance [72]. Although the mechanistic basis of this ‘background effect’ remains unclear, these results imply that caution must be used when extrapolating fitness measures of resistance in lab strains to clinical strains.

Clinical studies, on the other hand, have focused on documenting the dynamics of resistance without providing insight into the mechanisms that drive these patterns. For example, it is clear that resistance tends to decline in individual patients following antibiotic use. However it is unclear if these declines are caused by the reversion of resistance, the immigration of competitively superior antibiotic-sensitive strains, or by the spread of antibiotic-sensitive survivors of treatment. Whole-genome sequencing (WGS) of clinical isolates has started to provide invaluable insights into the evolution of pathogenic bacteria on a clinically relevant timescale [73–78], and large-scale sequencing of longitudinal samples of clinical isolates will provide a much more detailed view of the dynamics of resistance evolution. We suggest that it may be difficult to understand the significance of changes in the genetic structure of bacterial populations revealed by WGS unless sequencing is accompanied by phenotypic measurements of relevant traits, such as antibiotic resistance and competitive ability, which are commonly used in experimental evolution studies.

In summary, we think that progress in our understanding of the forces maintaining antibiotic resistance in pathogen populations will be best achieved using a combination of approaches from clinical microbiology and experimental evolution. It is important that we make progress in this area, because an increased understanding of the evolutionary processes that maintain resistance can be used to inform clinical usage of antibiotics. Much of the current effort in fighting antibiotic resistance is focussed on limiting antibiotic use via reduced prescriptions. If compensatory adaptation is highly pervasive, reducing antibiotic consumption is unlikely to drastically curtail antibiotic resistance. However we argue that compensatory adaptation is less prevalent in clinical real-world populations than previously thought, and therefore campaigns to reduce antibiotic usage should remain a major strategy for preventing antibiotic resistance.

## FUNDING

The research leading to these results has received funding from the European Research Council under the European Union's Seventh Framework Program (FP7/2007-2013)/ERC grant agreement no. 281591 and from the Royal Society.

**Conflict of interest**: None declared.

## References

[1] Andersson DI (2006). The biological cost of mutational antibiotic resistance: any practical conclusions?. Curr Opin Microbiol.

[2] Andersson DI, Hughes D (2010). Antibiotic resistance and its cost: is it possible to reverse resistance?. Nat Rev Microbiol.

[3] Baltrus DA (2013). Exploring the costs of horizontal gene transfer. Trends Ecol Evol.

[4] MacLean RC, Hall AR, Perron GG (2010). The population genetics of antibiotic resistance: integrating molecular mechanisms and treatment contexts. Nat Rev Genet.

[5] Levin BR, Perrot V, Walker N (2000). Compensatory mutations, antibiotic resistance and the population genetics of adaptive evolution in bacteria. Genetics.

[6] Lipsitch M, Bergstrom CT, Levin BR (2000). The epidemiology of antibiotic resistance in hospitals: paradoxes and prescriptions. Proc Natl Acad Sci U S A.

[7] zur Wiesch PA, Kouyos R, Engelstädter J (2011). Population biological principles of drug-resistance evolution in infectious diseases. Lancet Infect Dis.

[8] zur Wiesch PS, Engelstaedter J, Bonhoeffer S (2010). Compensation of fitness costs and reversibility of antibiotic resistance mutations. Antimicrob Agents Chemother.

[9] Tanaka MM, Valckenborgh F (2011). Escaping an evolutionary lobster trap: drug resistance and compensatory mutation in a fluctuating environment. Evolution.

[10] Schrag SJ, Perrot V (1996). Reducing antibiotic resistance. Nature.

[11] Schrag SJ, Perrot V, Levin BR (1997). Adaptation to the fitness costs of antibiotic resistance in *Escherichia coli*. Proc R Soc B Biol Sci.

[12] Maisnier-Patin S, Berg OG, Liljas L (2002). Compensatory adaptation to the deleterious effect of antibiotic resistance in *Salmonella typhimurium*. Mol Microbiol.

[13] Nagaev I, Bjorkman J, Andersson DI (2001). Biological cost and compensatory evolution in fusidic acid-resistant *Staphylococcus aureus*. Mol Microbiol.

[14] Comas I, Borrell S, Roetzer A (2012). Whole-genome sequencing of rifampicin-resistant *Mycobacterium tuberculosis* strains identifies compensatory mutations in RNA polymerase genes. Nat Genet.

[15] Farhat MR, Shapiro BJ, Kieser KJ (2013). Genomic analysis identifies targets of convergent positive selection in drug-resistant *Mycobacterium tuberculosis*. Nat Genet.

[16] de Vos M, Mueller B, Borrell S (2013). Putative compensatory mutations in the rpoC gene of rifampin-resistant *Mycobacterium tuberculosis* are associated with ongoing transmission. Antimicrob Agents Chemother.

[17] Costelloe C, Metcalfe C, Lovering A (2010). Effect of antibiotic prescribing in primary care on antimicrobial resistance in individual patients: systematic review and meta-analysis. Br Med J.

[18] Malhotra-Kumar S, Lammens C, Coenen S (2007). Effect of azithromycin and clarithromycin therapy on pharyngeal carriage of macrolide-resistant streptococci in healthy volunteers: a randomised, double-blind, placebo-controlled study. Lancet.

[19] Chung A, Perera R, Brueggemann AB (2007). Effect of antibiotic prescribing on antibiotic resistance in individual children in primary care: prospective cohort study. Br Med J.

[20] Sun L, Klein EY, Laxminarayan R (2012). Seasonality and temporal correlation between community antibiotic use and resistance in the United States. Clin Infect Dis.

[21] Dagan R, Barkai G, Givon-Lavi N (2008). Seasonality of antibiotic-resistant *Streptococcus pneumoniae* that causes acute otitis media: a clue for an antibiotic-restriction policy?. J Infect Dis.

[22] Enne VI, Livermore DM, Stephens P (2001). Persistence of sulphonamide resistance in *Escherichia coli* in the UK despite national prescribing restriction. Lancet.

[23] Sundqvist M, Geli P, Andersson DI (2010). Little evidence for reversibility of trimethoprim resistance after a drastic reduction in trimethoprim use. J Antimicrob Chemother.

[24] Enne VI, Bennett PM, Livermore DM (2004). Enhancement of host fitness by the sul2-coding plasmid p9123 in the absence of selective pressure. J Antimicrob Chemother.

[25] Bell BG, Schellevis F, Stobberingh E (2014). A systematic review and meta-analysis of the effects of antibiotic consumption on antibiotic resistance. BMC Infect Dis.

[39] Nielsen KL, Pedersen TM, Udekwu KI (2012). Fitness cost: a bacteriological explanation for the demise of the first international methicillin-resistant *Staphylococcus aureus* epidemic. J Antimicrob Chemother.

[40] Wielgoss S, Barrick JE, Tenaillon O (2011). Mutation rate inferred from synonymous substitutions in a long-term evolution experiment with *Escherichia coli*. G3.

[41] Gifford DR, MacLean RC (2013). Evolutionary reversals of antibtioic resistance in experimental populations of *Pseudomonas aeruginosa*. Evolution.

[42] Hall AR, Griffiths VF, MacLean RC (2010). Mutational neighbourhood and mutation supply rate constrain adaptation in Pseudomonas aeruginosa. Proc R Soc Lond B Biol Sci.

[43] Bjorkman J, Nagaev I, Berg OG (2000). Effects of environment on compensatory mutations to ameliorate costs of antibiotic resistance. Science.

[44] Meka VG, Gold HS, Cooke A (2004). Reversion to susceptibility in a linezolid-resistant clinical isolate of Staphylococcus aureus. J Antimicrob Chemother.

[45] Phillips G, Funnell B (2004). Plasmid Biology.

[46] Smith J (2001). The social evolution of bacterial pathogenesis. Proc R Soc Lond Biol Sci.

[47] Vogwill T, MacLean RC (2015). The genetic basis of the fitness cost of antimicrobial resistance: a meta-anlysis approach. Evol Appl..

[48] Drusano GL (2004). Antimicrobial pharmacodynamics: critical interactions of ‘bug and drug’. Nat Rev Microbiol.

[49] Balaban NQ (2011). Persistence: mechanisms for triggering and enhancing phenotypic variability. Curr Opin Genet Dev.

[50] Balaban NQ, Merrin J, Chait R (2004). Bacterial persistence as a phenotypic switch. Science.

[51] Javid B, Sorrentino F, Toosky M (2014). Mycobacterial mistranslation is necessary and sufficient for rifampicin phenotypic resistance. Proc Natl Acad Sci U S A.

[52] Silander OK, Nikolic N, Zaslaver A (2012). A genome-wide analysis of promoter-mediated phenotypic noise in *Escherichia coli*. PLoS Genet.

[53] Avery SV (2006). Microbial cell individuality and the underlying sources of heterogeneity. Nat Rev Microbiol.

[54] Sánchez-Romero MA, Casadesús J (2013). Contribution of phenotypic heterogeneity to adaptive antibiotic resistance. Proc Natl Acad Sci U S A.

[55] Ni M, Decrulle AL, Fontaine F (2012). Pre-disposition and epigenetics govern variation in bacterial survival upon stress. PLoS Genet.

[56] Wakamoto Y, Dhar N, Chait R (2013). Dynamic persistence of antibiotic-stressed *Mycobacteria*. Science.

[57] Zhang H, Li D, Zhao L (2013). Genome sequencing of 161 *Mycobacterium tuberculosis* isolates from China identifies genes and intergenic regions associated with drug resistance. Nat Genet.

[58] Brandis G, Wrande M, Liljas L (2012). Fitness-compensatory mutations in rifampicin-resistant RNA polymerase. Mol Microbiol.

[59] Maisnier-Patin S, Andersson DI (2004). Adaptation to the deleterious effects of antimicrobial drug resistance mutations by compensatory evolution. Res Microbiol.

[60] Löfmark S, Jernberg C, Billström H (2008). Restored fitness leads to long-term persistence of resistant Bacteroides strains in the human intestine. Anaerobe.

[61] Karami N, Hannoun C, Adlerberth I (2008). Colonization dynamics of ampicillin-resistant *Escherichia coli* in the infantile colonic microbiota. J Antimicrob Chemother.

[62] Sjölund M, Wreiber K, Andersson DI (2003). Long-term persistence of resistant enterococcus species after antibiotics to eradicate *Helicobacter pylori*. Ann Intern Med.

[63] World Health Organization (2014). Antimicrobial resistance: global report on surveillance 2014.

[64] Kumarasamy KK, Toleman MA, Walsh TR (2010). Emergence of a new antibiotic resistance mechanism in India, Pakistan, and the UK: a molecular, biological, and epidemiological study. Lancet Infect Dis.

[65] Hawkey PM, Jones AM (2009). The changing epidemiology of resistance. J Antimicrob Chemother.

[66] Alekshun MN, Levy SB (2007). Molecular mechanisms of antibacterial multidrug resistance. Cell.

[67] Levy SB, Marshall B (2004). Antibacterial resistance worldwide: causes, challenges and responses. Nat Med.

[68] Carattoli A (2013). Plasmids and the spread of resistance. Int J Med Microbiol.

[69] Harrison E, Brockhurst MA (2012). Plasmid-mediated horizontal gene transfer is a coevolutionary process. Trends Microbiol.

[70] Millan AS, Peña-Miller R, Toll-Riera M (2014). Positive selection and compensatory adaptation interact to stabilize non-transmissible plasmids. Nat Commun.

[71] Bouma JE, Lenski RE (1988). Evolution of a bacteria/plasmid association. Nature.

[72] Vogwill T, Kojadinovic M, Furió V (2014). Testing the role of genetic background in parallel evolution using the comparative experimental evolution of antibiotic resistance. Mol Biol Evol.

[73] Smith EE, Buckley DG, Wu Z (2006). Genetic adaptation by *Pseudomonas aeruginosa* to the airways of cystic fibrosis patients. Proc Natl Acad Sci U S A.

[74] Croucher NJ, Harris SR, Fraser C (2011). Rapid pneumococcal evolution in response to clinical interventions. Science.

[75] Mwangi MM, Wu SW, Zhou Y (2007). Tracking the in vivo evolution of multidrug resistance in *Staphylococcus aureus* by whole-genome sequencing. Proc Nat Acad Sci U S A.

[76] Lieberman TD, Michel J-B, Aingaran M (2011). Parallel bacterial evolution within multiple patients identifies candidate pathogenicity genes. Nat Genet.

[77] Young BC, Golubchik T, Batty EM (2012). Evolutionary dynamics of *Staphylococcus aureus* during progression from carriage to disease. Proc Natl Acad Sci U S A.

[78] Marvig RL, Johansen HK, Molin S (2013). Genome analysis of a transmissible lineage of *Pseudomonas aeruginosa* reveals pathoadaptive mutations and distinct evolutionary paths of hypermutators. PLoS Genet.

